# Changes in the proportion of clinical clusters contribute to the phenotypic evolution of Behçet’s disease in Japan

**DOI:** 10.1186/s13075-020-02406-6

**Published:** 2021-02-01

**Authors:** Yutaro Soejima, Yohei Kirino, Mitsuhiro Takeno, Michiko Kurosawa, Masaki Takeuchi, Ryusuke Yoshimi, Yumiko Sugiyama, Shigeru Ohno, Yukiko Asami, Akiko Sekiguchi, Toshihisa Igarashi, Shohei Nagaoka, Yoshiaki Ishigatsubo, Hideaki Nakajima, Nobuhisa Mizuki

**Affiliations:** 1 Department of Stem Cell and Immune Regulation, Yokohama City University Graduate School of Medicine, 3-9 Fukuura, Kanazawa-ku, Yokohama, 236-0004 Japan; 2 Department of Allergy and Rheumatology, Nippon Medical School, Musashi Kosugi Hospital, 1-396 Kosugi-machi, Nakahara-ku, Kawasaki, Kanagawa 211-8533 Japan; 3 Department of Epidemiology and Environmental Health, Juntendo University Faculty of Medicine, 2-1-1 Hongo, Bunkyo-Ku, Tokyo, 113-0033 Japan; 4 Department of Ophthalmology and Visual Science, Yokohama City University Graduate School of Medicine, 3-9 Fukuura, Kanazawa-ku, Yokohama, 236-0004 Japan; 5 Yokohama City University Medical Center, Center for Rheumatic Diseases, 4-57 Urafunecho, Minami-ku, Yokohama, 232-0024 Japan; 6 Yokosuka Center for Rheumatic Diseases, Yokosuka City Hospital, 1-3-2 Nagasaka, Yokosuka, 240-0101 Japan; 7 Department of Hematology and Rheumatology, Fujisawa City Hospital, 2-6-1 Fujisawa, Fujisawa, 251-8550 Japan; 8 Department of Rheumatology, Yamato City Hospital, Fukaminishi, Yamato, 242-8602 Japan; 9 Department of Rheumatology, Yokohama Minami Kyosai Hospital, 1-21-1 Mutsuura Higashi, Yokohama, 236-0037 Japan; 10 Yokohama City University, Yokohama, 3-9 Fukuura, Kanazawa-ku, Yokohama, 236-0004 Japan

**Keywords:** Behçet’s disease, Cluster analysis, Intestinal manifestation

## Abstract

**Background:**

We hypothesized that Behçet’s disease (BD) consists of several clinical subtypes with different severity, resulting in heterogeneity of the disease. Here, we conducted a study to identify clinical clusters of BD.

**Methods:**

A total of 657 patients registered in the Yokohama City University (YCU) regional BD registry between 1990 and 2018, as well as 6754 patients who were initially registered in the Japanese Ministry of Health, Labour and Welfare (MHLW) database between 2003 and 2014, were investigated. The YCU registry data regarding the clinical manifestations of BD, human leukocyte antigen (HLA) status, treatments, and hospitalizations were analyzed first, followed by similar analyses of the MHLW for validation. A hierarchical cluster analysis was independently performed in both patient groups.

**Results:**

A hierarchical cluster analysis determined five independent clinical clusters in the YCU cohort. Individual counterparts of the YCU clusters were confirmed in the MHLW registry. Recent phenotypical evolutions of BD in Japan, such as increased gastrointestinal (GI) involvement, reduced complete type according to the Japan Criteria, and reduced HLA-B51 positivity were associated with chronologically changing proportions of the clinical clusters.

**Conclusions:**

In this study, we identified independent clinical clusters among BD patients in Japan and found that the proportion of each cluster varied over time. We propose five independent clusters namely “mucocutaneous”, “mucocutaneous with arthritis”, “neuro”, “GI”, and “eye.”

## Background

Behçet’s disease (BD) is a rare systemic inflammatory disease that causes periodic ocular and mucocutaneous inflammation [1, 2]. Environmental and genetic factors such as human leukocyte antigen (HLA)-B51 have been implicated in its pathogenesis [3–5]. The 2018 update EULAR recommendation emphasized individualized treatment for BD [6]: intensive treatment is particularly necessary for ocular, vascular, neurological, and gastrointestinal (GI) symptoms, which are associated with poor outcomes [6, 7]. Therefore, identifying predictive factors for these unfavorable BD subtypes is essential.

One of the primary obstacles facing personalized medicine is the heterogeneity of BD patients [2]. Serious organ involvement such as ocular and vascular lesions in young men [8, 9], genital ulcers in young women [9], and GI symptoms in pediatric patients [9, 10] have been reported. Geographic and genetic disease variations are other features of BD, including distinct clinical manifestations in countries with prevalent disease [1, 2, 11]. GI involvement is more frequent in Far East-Asian countries, including Japan [9, 12–14], whereas vascular involvement is more common in Mediterranean and Middle Eastern countries [15, 16].

The clinical features of BD have evolved in Japan despite the preservation of a homogenous genetic background because of low immigration rates. Increased GI manifestations, decreased eye involvement, and fewer HLA-B51-positive patients over the past 3 decades have been reported [13, 17, 18]. The same trend was observed in Korea, where GI involvement is also prevalent [14]. Interestingly, an explosive increase was also observed in inflammatory bowel diseases in East Asian countries [19, 20]. It is plausible that the phenotypic changes are caused by preferential expansion of a particular subset of BD in Japan; this idea is supported by previous meta-analyses that revealed a strong association between HLA-B51 and both eye involvement and male sex, whereas its association with GI involvement was weak [21, 22].

Because of the diverse manifestations of this disease, we aimed to stratify patients with varying clinical manifestations and different prognoses. For example, the genetic structures of anti-citrullinated protein antibody-positive and -negative patients with rheumatoid arthritis are different; an HLA-shared epitope is more common in patients of the former group, who also experience progressive bone destruction [23–25]. Previous studies have shown that patients with BD are categorized into several distinct phenotypic clusters associated with varying prognoses [26–29]. Clustering analyses have delineated several possible BD subsets, including vascular disease, while another subset is an acne-arthritis-enthesitis cluster in Turkey [26]. Interestingly, the proposed acne-arthritis-enthesitis cluster showed familial aggregation, suggesting a possible contribution of subset-specific genetic factors [30].

Prognostic factors for BD are necessary, but the disease heterogeneity is precluding the development of precision medicine. In this study, clustering analysis was performed on patients with BD using data from nationwide and regional registries in Japan to stratify complex phenotype of BD.

## Methods

### Setting

Data from patients with BD treated in Japan were analyzed retrospectively. All patients met the 1987 revised diagnostic criteria for BD as designated by the Behçet’s Disease Research Committee (BDRC) of Japan [31]. According to Japanese BD diagnostic criteria, oral ulcers, skin lesions, eye inflammation, and genital ulcers are “major symptoms;” arthritis, epididymitis, gastrointestinal symptoms, vascular lesions, and neurological manifestations are “minor symptoms.” Patients in one of the following categories meet criteria for BD: (1) all four major symptoms, (2) three major symptoms, (3) two major and two minor symptoms, (4) typical recurrent ocular inflammation and one or more major symptoms, or (5) typical recurrent ocular inflammation and two minor symptoms meet criteria for BD diagnosis. “GI symptoms” were noted only for patients who were found to have deep ulcers in the ileocecum area, whereas patients with “neurological manifestations” included those with parenchymal neuro-BD.

To minimize patient heterogeneity and increase comparability with other studies, we also applied the International Study Group (ISG) and International Team for the Revision of the International Criteria for Behçet’s Disease (ITR-ICBD) criteria [32, 33].

### Hospital-based regional BD registry

The Yokohama City University (YCU) registry includes BD patients who were treated at any of the seven hospitals within the Kanagawa district in mid-Japan from July 1991 to December 2018. All the patients were Japanese except eight patients (one Filipino, one Syrian, one Korean, two Chinese, three dual heritage of Pakistani, Finnish, and French). Retrospectively, we obtained the following data: date of birth, sex, date of BD onset, follow-up period, individual symptoms of BD, HLA-B51 positivity, episode of hospitalization, blindness, death caused by BD, and medications. Blindness is defined as visual acuity of less than 3/60, or a corresponding visual field loss to less than 10°, even with just one eye.

### Japan national BD registry

Since 1972, the Japan MHLW has been granted public subsidies for patients’ medical expenses for treatment of intractable diseases, including BD. To be eligible for this assistance, patients must meet the BRDC criteria and provide documentation of their date of birth, gender, date of onset, the presence of BD symptoms included in the Japanese BD diagnostic criteria (as filed by the attending physician), questions about laboratory data, admission episodes, and medical treatment. We extracted data recorded in the MHLW national registry between 2003 and 2014; to adjust for the observation period, only newly submitted cases were included. We selected newly registered patients in order to adjust for the observation period, given that manifestations can accumulate over one’s clinical course and change the BD-associated phenotype [17]. Moreover, the effects of treatment on the phenotypes were marginal in this group because new registrations were linked to financial support regarding medical costs. Some of the patients were diagnosed before 2003 although they were initially registered after that year.

### Statistical analysis

R version 3.6.2 (http://cran.r-project.org/) was used for our analyses. We performed Ward’s hierarchical cluster analysis with dissimilarity measures of the square Euclidean distance for both registry data. The validity of the number of clusters was assessed by several methods based on inter- and intra-cluster “scatter” indicators [34–36]. In these methods, the indices CH, indicator diffH, and indicator KL/minimization indicator H, which maximizes the number of clusters, were selected as arguments. The Kruskal-Wallis and chi-squared were performed to compare groups. A *P* value < 0.05 was considered statistically significant.

## Results

### Participants

Among 707 patients with BD in the YCU registry and 8282 in the MHLW registry, 657 and 6754 patients were included after excluding those having missing data (Supplementary Figure 1). Table 1 shows the clinical characteristics of the two patient groups. These characteristics were similar to those previously reported in Japan, albeit with a lower incidence of eye involvement in the MHLW registry [1].

**Table 1:** Characteristic of patients with Behçet’s disease who were enrolled in the study

Characteristics	Yokohama City University registry (***n*** = 657)	Japan national registry (***n*** = 6754)
Sex: female/male, *n* (%)	372/285 (56.6/43.4)	3990/2764 (59.1/40.9)
Age of diagnosis (mean ± SD)	36.58 ± 12.29	36.68 ± 14.23
Observation period (mean ± SD)	13.72 ± 11.79	4.14 ± 7.62
*Disease phenotype*		
Oral ulcer, *n* (%)	653 (99.4)	6336 (93.8)
Skin involvement, *n* (%)	585 (89.0)	5483 (81.2)
Eye involvement, *n* (%)	392 (59.7)	2344 (34.7)
Genital ulcer, *n* (%)	474 (72.1)	4242 (62.8)
Arthritis, *n* (%)	346 (52.7)	3311 (49.0)
Epididymitis, *n*/available data number (%)	15/285 (5.3)	257/2764 (9.3)
Gastrointestinal involvement, *n* (%)	113 (17.2)	906 (13.4)
Vascular involvement, *n* (%)	55 (8.4)	148 (2.2)
Neurological involvement, *n* (%)	67 (10.2)	350 (5.2)
Pathergy test, *n*/available data number (%)	74/170 (43.5)	1361/4225 (32.2)
*Other characteristics*		
HLA-B51, *n*/available data number (%)	214/449 (47.7)	1389/3141 (44.2)
Fulfilling ISG criteria, *n* (%)	583 (88.7)	4980 (73.7)
Fulfilling ITR-ICBD criteria, *n* (%)	645 (98.2)	5586 (82.7)
Smoking, *n*/available data number (%)	233/455 (51.2)	N/A
Hospitalization, *n*/available data number (%)	239/522 (45.8)	1556/6002 (25.9)
Blindness, *n*/available data number (%)	98/582 (16.8)	N/A
Death caused by BD, *n*/available data number (%)	3/520 (0.6)	N/A
*Treatments*		
Colchicine, *n* (%)	375/522 (71.8)	2942 (43.6)
Glucocorticoid, *n* (%)	236/522 (45.2)	2352 (34.8)
Maximum Prednisolone dose (mg/day, mean ± SD)	11.75 ± 17.76	N/A
Methylprednisolone pulse therapy, *n*/available data number (%)	36/522 (6.9)	N/A
Immunosuppressant therapy, *n*/available data number (%)	203/522 (38.9)	578 (8.6)
Biologics, *n* (%)	95 (14.5)	N/A
Infliximab, *n* (%)	86 (13.1)	N/A
Adalimumab, *n* (%)	24 (3.7)	N/A
Others, *n* (%)*	3 (0.5)	N/A
Time from diagnosis to biologics (years, mean ± SD)	6.22 ± 8.52	N/A

*BDRC* Behçet’s Disease Research Committee, *ISG* International Study Group, *ITR-ICBD* International Team for the Revision of the International Criteria for Behçet’s Disease, *N/A* not available

*Others: etanercept 2, tocilizumab 1

### Discovery of distinct clusters in the hospital-based BD registry

We first performed cluster analysis using 8 variables (4 major and 4 minor symptoms as described in the Japanese criteria except for epididymitis). A dendrogram revealed 5 major clusters assigned as clusters 1 to 5 (Supplementary Figure 2a). The validity of the number of clusters was supported by statistical analysis (Supplementary Table 1). We then compared the clinical features of each cluster (Table 2). In brief, all patients in cluster 1 showed mucocutaneous symptoms without vital organ involvement except in the eyes. Cluster 2 was characterized by the highest proportion of GI involvement (75.7%), and the lowest of eye involvement (34.3%). In cluster 3, mucocutaneous symptoms without vital organ involvement were main features like cluster 1, but arthritis was absent. In cluster 4, all patients had eye involvement with low frequency of other mucocutaneous manifestations. Cluster 5 was associated with high rates of neurological involvement (98.5%).

**Table 2:** Characteristics of the cluster with Behçet’s Disease patients in Yokohama City University registry

Characteristics	Total (***n*** = 657)	Cluster 1 (***n*** = 164)	Cluster 2 (***n*** = 140)	Cluster 3 (***n*** = 140)	Cluster 4 (***n*** = 146)	Cluster 5 (***n*** = 67)	***P***
*Clinical symptoms used in clustering*							
Oral ulcer, *n* (%)	653 (99.4)	164 (100.0)	138 (98.6)	140 (100.0)	144 (98.6)	67 (100.0)	0.265
Skin involvement, *n* (%)	585 (89.0)	164 (100.0)	130 (92.9)	140 (100.0)	93 (63.7)	58 (86.6)	< 0.001
Eye involvement, *n* (%)	392 (59.7)	78 (47.6)	48 (34.3)	76 (54.3)	146 (100.0)	44 (65.7)	< 0.001
Genital ulcer, *n* (%)	474 (72.1)	164 (100.0)	105 (75.0)	140 (100.0)	21 (14.4)	44 (65.7)	< 0.001
Arthritis, *n* (%)	346 (52.7)	164 (100.0)	94 (67.1)	0 (0.0)	57 (39.0)	31 (46.3)	< 0.001
Gastrointestinal involvement, *n* (%)	113 (17.2)	0 (0.0)	106 (75.7)	0 (0.0)	0 (0.0)	7 (10.4)	< 0.001
Vascular involvement, *n* (%)	55 (8.4)	0 (0.0)	54 (38.6)	0 (0.0)	0 (0.0)	1 (1.5)	< 0.001
Neurological involvement, *n* (%)	67 (10.2)	0 (0.0)	1 (0.7)	0 (0.0)	0 (0.0)	66 (98.5)	< 0.001
*Criteria and HLA typing, smoking, and hospitalization rates*							
Sex: female/male, *n*/available data number (%)	372/285 (56.6/43.4)	124/40 (75.6/24.4)	80/60 (57.1/42.9)	90/50 (64.3/35.7)	46/100 (31.5/68.5)	32/35 (47.8/52.2)	< 0.001
Age at onset (years, mean ± SD)	36.58 ± 12.29	37.37 ± 12.25	35.41 ± 13.41	33.59 ± 10.25	40.48 ± 12.84	34.79 ± 10.43	< 0.001
Observation period (years, mean ± SD)	13.72 ± 11.79	16.33 ± 12.07	13.72 ± 12.52	13.27 ± 12.53	10.16 ± 9.44	16.08 ± 10.70	< 0.001
Pathergy test, *n* (%)	74/170 (43.5)	27/66 (40.9)	7/23 (31.8)	21/34 (61.8)	13/32 (40.6)	6/16 (37.5)	0.174
HLA-B51, *n* (%)	214/449 (47.7)	58/114 (50.9)	29/88 (33.0)	49/94 (52.1)	49/98 (50.0)	29/55 (52.7)	0.047
Fulfilling ISG criteria, *n* (%)	583 (88.7)	164 (100.0)	107 (76.4)	140 (100.0)	113 (77.4)	59 (88.1)	< 0.001
Fulfilling ITR-ICBD criteria, *n* (%)	645 (98.2)	164 (100.0)	128 (91.4)	140 (100.0)	146 (100.0)	67 (100.0)	< 0.001
Hospitalization, *n*/available data number (%)	239/522 (45.8)	35/115 (30.4)	69/97 (71.1)	43/128 (33.6)	49/125 (39.2)	43/57 (75.4)	< 0.001
Blindness, *n*/available data number (%)	98/582 (16.8)	10/141 (7.1)	8/124 (6.5)	22/133 (16.5)	34/123 (27.6)	24/61 (39.3)	< 0.001
Death caused by BD, n/available data number (%)	3/520 (0.6)	0/114 (0.0)	2/96 (2.1)	1/128 (0.8)	0/125 (0.0)	0/57 (0.0)	0.230
*Treatments*							
Colchicine, *n*/available data number (%)	375/522 (71.8)	73/115 (63.5)	77/97 (79.4)	83/128 (64.8)	96/125 (76.8)	46/57 (80.7)	0.009
Glucocorticoids, *n*/available data number (%)	236/522 (45.2)	40/115 (34.8)	65/97 (67.0)	44/128 (34.4)	42/125 (33.6)	45/57 (78.9)	< 0.001
Maximum dose of prednisolone (mg/day, mean ± SD)	11.75 ± 17.76	5.64 ± 11.15	18.98 ± 19.77	6.81 ± 12.71	8.16 ± 14.24	31.81 ± 23.64	< 0.001
Methylprednisolone pulse therapy, *n*/available data number (%)	36/522 (6.9)	2/115 (1.7)	9/97 (9.3)	2/128 (1.6)	4/125 (3.2)	19/57 (33.3)	< 0.001
Immunosuppressants, *n*/available data number (%)	203/522 (38.9)	17/115 (14.8)	71/97 (73.2)	30/128 (23.4)	47/125 (37.6)	38/57 (66.7)	< 0.001
Biologics, *n* (%)	95 (14.5)	9 (5.5)	23 (16.4)	15 (10.7)	34 (23.3)	14 (20.9)	< 0.001
Time from diagnosis to biologics (years, mean ± SD)	6.22 ± 8.52	4.86 ± 6.90	7.47 ± 10.95	10.07 ± 10.20	2.96 ± 4.46	8.54 ± 8.42	0.043

*ISG* International Study Group, *ITR-ICBD* International Team for the Revision of the International Criteria for Behçet’s Disease

We compared other features including demography, HLA-B51 positivity, fulfillment of the ISG and the ITR-ICBD criteria, therapy, and hospitalization events among the 5 clusters. More female patients were found in clusters 1 and 3, whereas male was predominant in cluster 4. Frequency of HLA-B51-positive individuals was comparable among clusters, except cluster 2 (GI cluster) that showed significantly lower positivity (33.0%) than any other clusters. Less than quarter of patients did not meet the ISG criteria in clusters 2, 4, and 5, whereas all patients met the ITR-ICBD criteria except 12 patients (8.6%) in cluster 2. However, background characteristics of individual clusters were unchanged in patients meeting the ISG or ITR-ICBD criteria (Supplementary Tables 2 and 3). Besides clinical manifestations, the data showed therapeutic approaches were different among the clusters. The use of glucocorticoids, particularly high dosage, and immunosuppressants were more common in cluster 2 and cluster 5 than the others, due to the gastrointestinal and neurological involvement, respectively. Introduction of TNF inhibitors was the most common and the earliest in cluster 4 because of the eye involvement (periods from the diagnosis to administration; 2.96 ± 4.46 years). The cluster was associated with low incidence of mucocutaneous symptoms, and high unsatisfactory rate of ISG criteria (22.3%). On the other hand, patients in clusters 2 and 5 needed frequent hospitalization compared with the others, suggesting that they had more severe physical condition. Blindness is more in clusters 4 and 5, but most cases with blindness were diagnosed before 1999 (Supplementary Table 4). We thought that it is due to the advance of therapy, such as TNF inhibitors. The number of death caused by BD was very small (3 cases).

### Validation in the Japanese national BD registry

To validate the findings, the same clustering analysis was conducted using the MHLW dataset (Supplementary Figure 2b). Although approximately 200 patients in the YCU registry overlapped with the MHLW database, these patients were not excluded because of complete anonymization. A hierarchical cluster analysis of eight BD clinical manifestations identified seven different clusters that were statistically valid (Supplementary Table 1) and designated as clusters A to G (Table 3). The clustering patterns between the YCU Registry and the MHLW database corresponded to clusters 1 and E, clusters 2 and A, clusters 3 and G, clusters 4, D and F, and clusters 5 and B, respectively, but there was some discrepancy. The exception was cluster C, which had the lowest frequency of oral ulcers, suggesting that the MHLW database may contain suspicious BD cases. Otherwise, the features of each cluster in the MHLW database were also shared with counterparts of the clusters in the YCU registry. For example, cluster A in the MHLW database was characterized by the frequent GI and uncommon eye involvement, low HLA-B51 positivity, low fulfillment of the ISG and ITR-ICBD criteria, and high frequency of hospitalization. These characteristics were also found in the counterpart cluster, cluster 2 in the YCU registry.

**Table 3:** Characteristics of the cluster with Behçet’s disease patients in MHLW registry

	Total (***n*** = 6754)	Cluster A (***n*** = 1131)	Cluster B (***n*** = 876)	Cluster C (***n*** = 689)	Cluster D (***n*** = 805)	Cluster E (***n*** = 1369)	Cluster F (***n*** = 799)	Cluster G (***n*** = 1085)	***P***
**Clinical symptoms used in clustering**									
Oral ulcer, *n* (%)	6336 (93.8)	1050 (92.8)	867 (99.0)	361 (52.4)	805 (100.0)	1369 (100.0)	799 (100.0)	1085 (100.0)	< 0.001
Skin involvement, *n* (%)	5483 (81.2)	493 (43.6)	755 (86.2)	177 (25.7)	805 (100.0)	1369 (100.0)	799 (100.0)	1085 (100.0)	< 0.001
Eye involvement, *n* (%)	2344 (34.7)	87 (7.7)	115 (13.1)	538 (78.1)	805 (100.0)	0 (0.0)	799 (100.0)	0 (0.0)	< 0.001
Genital ulcer, *n* (%)	4242 (62.8)	575 (50.8)	202 (23.1)	212 (30.8)	0 (0.0)	1369 (100.0)	799 (100.0)	1085 (100.0)	< 0.001
Arthritis, *n* (%)	3311 (49.0)	461 (40.8)	484 (55.3)	192 (27.9)	390 (48.4)	1369 (100.0)	415 (51.9)	0 (0.0)	< 0.001
Gastrointestinal involvement, *n* (%)	906 (13.4)	879 (77.7)	0 (0.0)	27 (3.9)	0 (0.0)	0 (0.0)	0 (0.0)	0 (0.0)	< 0.001
Vascular involvement, *n* (%)	148 (2.2)	0 (0.0)	121 (13.8)	27 (3.9)	0 (0.0)	0 (0.0)	0 (0.0)	0 (0.0)	< 0.001
Neurological involvement, *n* (%)	350 (5.2)	0 (0.0)	289 (33.0)	61 (8.9)	0 (0.0)	0 (0.0)	0 (0.0)	0 (0.0)	< 0.001
**Other characteristics**									
Sex: female/male, *n* (%)	3990/2764 (59.1/40.9)	654/477 (57.8/42.2)	427/449 (48.7/51.3)	299/390 (43.4/56.6)	295/510 (36.6/63.4)	1032/337 (75.4/24.6)	479/320 (59.9/40.1)	804/281 (74.1/25.9)	< 0.001
Age at onset (years, mean ± SD)	36.68 ± 14.23	39.91 ± 17.73	38.54 ± 14.46	38.78 ± 15.14	36.82 ± 13.09	35.58 ± 12.64	34.45 ± 12.00	33.38 ± 12.37	< 0.001
Observation period (years, mean ± SD)*	4.14 ± 7.62	4.05 ± 7.71	4.18 ± 7.28	6.32 ± 9.95	3.82 ± 6.91	3.07 ± 6.06	6.19 ± 9.90	2.87 ± 5.57	< 0.001
Pathergy test, *n*/available data number (%)	1361/4225 (32.2)	225/706 (31.9)	171/536 (31.9)	117/431 (27.1)	149/404 (29.6)	295/860 (34.3)	175/508 (34.4)	229/680 (33.7)	0.099
HLA-B51, *n*/available data number (%)	1389/3141 (44.2)	172/513 (33.5)	237/492 (48.0)	172/358 (48.0)	221/413 (53.5)	244/599 (40.7)	164/321 (51.1)	179/443 (40.4)	< 0.001
Fulfilling ISG criteria, *n* (%)	4980 (73.7)	405 (35.8)	333 (38.0)	185 (26.9)	805 (100.0)	1368 (99.9)	799 (100.0)	1085 (100.0)	< 0.001
Fulfilling ITR-ICBD criteria, *n* (%)	5586 (82.7)	657 (58.1)	456 (52.1)	416 (60.4)	805 (100.0)	1368 (99.9)	799 (100.0)	1085 (100.0)	< 0.001
Recent hospitalization, *n*/available data number (%)**	1556/6002 (25.9)	400/971 (41.2)	299/771 (38.8)	144/625 (23.0)	77/738 (10.4)	271/1195 (22.7)	121/728 (16.6)	244/974 (25.1)	< 0.001

*ISG* International Study Group, *ITR-ICBD* International Team for the Revision of the International Criteria for Behçet’s Disease

*“Observation period” indicates the period from disease onset to application of specific disease by the Japanese government

**“Recent hospitalization” indicates episode of admission less than 6 months per patients

Thus, our data showed that clusters in the hospital-based BD registry were reproduced in the nationwide registry in which patients with a short disease duration were enriched, indicating that the clustering patterns represent those in BD patients in Japan.

### Chronological changes of clinical clusters

We previously reported that Japanese BD patient phenotypes have been evolving over the past 30 years, with ocular involvement becoming less common while the incidence of intestinal BD continues to rise [9, 13]. Similar evolution of clinical phenotypes has also been reported in Korea [14]. In Japan, the phenotypical changes were also associated with decreased HLA-B51 positivity in the patients [9, 13]. Therefore, we hypothesized that the recent phenotypic evolution of BD is caused by alternating proportion of individual disease clusters in Japan.

**Fig. 1:**
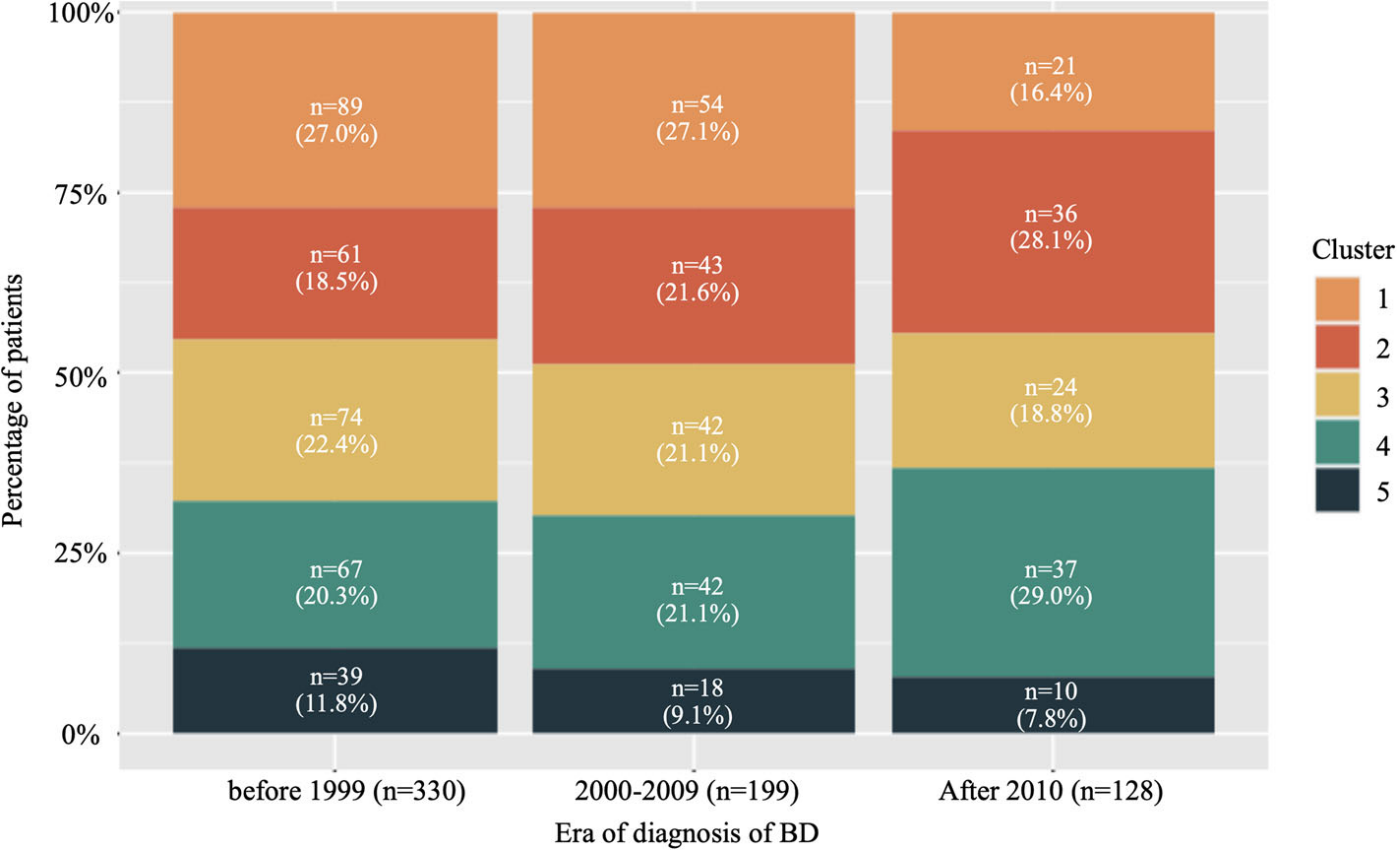
Evolution of clusters in the Yokohama City University registry. The *Y*-axis represents the percentage of each cluster to the total number of specified periods

As in a previous study [13], we compared the proportion of clusters in the three generation groups according to the year of onset in the YCU registry. We found a continuous increase in clusters 2 and 4, but a gradual decrease in the other clusters (Fig. 1). Particularly, expansion of cluster 2 characterized by GI involvement and low HLA-B51 positivity is concordant with recent epidemiological changes in Japan [13]. Another increment was found in cluster 4, which was characterized by the most common and earliest use of TNF inhibitors and a lower incidence of mucocutaneous symptoms (Table 2), particularly after 2010 (Fig. 1). Proportions of the other clusters had been relatively shrinking.

## Discussion

Because BD involves variable disease manifestations, many investigators refer to it as “Behçet’s syndrome” [6, 28]. Our data strongly support the notion that BD patients can be categorized into several clinical subtypes based on clinical presentations. A hierarchical clustering analysis of the hospital-based, YCU registry identified five clinical clusters, which consist of “mucocutaneous,” “mucocutaneous with arthritis,” “neuro,” “GI,” and “eye without other involvements” subtypes. The similar clustering pattern was also reproduced in the national registry, the MHLW database. Moreover, the present data showed that recent phenotypical evolution of BD was associated with altering the proportion of individual clinical clusters in the YCU registry. The chronological proportional changes of clinical subtypes were considered to cause recent epidemiological changes of BD patients in Japan, which are characterized by an increase in GI involvement and decreases complete type according to the Japan Criteria and HLA-B51 positivity.

Both genetic and environmental factors have been implicated in the formation of clinical clusters. However, due to the relative stability of the genetic background in Japan, recent changes in the clinical picture of BD patients over time may be dependent solely on environmental factors. Chronological analysis of the YCU registry showed two clinical clusters which had been recently expanded: one is cluster 2, which is a cluster with the highest proportion of GI patients and has expanded over the past 30 years in the YCU registry. Counterpart of the GI cluster was considered to correspond to cluster A in the MHLW database. The GI involvement is much more frequent in patients in East Asian countries including Japan and Korea than any regions, but it is unclear what is responsible for the geographical concentration. In addition to similar genetic backgrounds, the preferential use of the Japanese BD diagnostic criteria in these 2 countries [31], which incorporate the intestinal symptoms, is a possible reason. Interestingly, a westernized lifestyle is considered to contribute to recent rapid increase in inflammatory bowel diseases such as Crohn disease and ulcerative colitis in both countries [20]. Likewise, environmental changes might be involved in recent expansion of the GI cluster in BD patients in the East Asia.

In the YCU, another expansion was found in cluster 4, which was characterized by the highest incidence of, eye involvement, low incidence of mucocutaneous symptoms, and most common and earliest use of TNF inhibitors. The total number of patients with ocular lesions has decreased (Supplementary Table 4), but cluster 4 has increased. We consider that it is because TNF inhibitors for ocular involvement have a decreasing effect on mucocutaneous symptoms. This cluster was considered to correspond to clusters D and F in the MHLW database. Unlike the GI cluster, the cluster expansion was prominent after 2010. This is not likely coincident, because the use of TNF inhibitors was rapidly increased after official approval of infliximab and adalimumab for the BD related uveitis at 2007 and 2016, respectively, in Japan. Notably, the YCU has a Behçet’s Disease Medical Research Center with experienced rheumatologists and ophthalmologists, prompting this trend because ophthalmology department of YCU had many severe BD uveitis patients and they were administered TNF inhibitors. As disease modifying effects have been shown with early introduction of azathioprine [37], it is plausible that early and intensive treatments including biologics for the ocular involvement suppress extraocular manifestations, resulting in low incidence of mucocutaneous symptoms and low satisfactory rate of the ISG criteria in the cluster. However, besides the treatment related disease modifying effects, other factors also contribute to the formation of this cluster, because substantial number of patients was categorized into the cluster in the pre-biologics era. On the other hand, clusters 1, 3, and 5, identified in the YCU registry, are steadily shrinking in Japan (Fig. 1). These clusters include patients with the ocular and neurological involvement. Besides, a recent study also found that ocular disease is becoming milder in Japan [38], though the reason remains uncertain.

A study from Japan has shown that neurological involvement is more likely to develop in male smokers [39], suggesting that the recent shrinkage in cluster 5 is relate to fewer male smokers in Japan, though positive or negative effects of smoking on BD have been controversial [39–42]. Alternatively, improvement of oral hygiene [43] and altered microbiota [44] can also contribute to lowering the rate of serious BD phenotypes. It is important to determine environmental factors that are alterable unlike genetic backgrounds, especially when considering prophylactic strategies.

Most of the findings were consistent between the YCU and MHLW databases, but there were some discrepancies. For example, cluster 2 of the YCU had a high frequency of complications of GI and vascular involvements [2], but cluster A of the MHLW database did not show a significant association between the two. We suspect that different periods of observation are one of the reasons for this discrepancy (YCU, 13.7 ± 11.8 years; MHLW, 4.1 ± 7.6 years). In addition, while data were collected cross-sectionally in the MHLW, data were accumulated over time in the YCU registry. As shown in previous studies, symptoms of BD accumulate over time, and therefore, additional symptoms are likely to appear after the end of the observation period in the MHLW database [13, 17]. In fact, the incidence of GI and vascular lesions differed, especially vascular lesions, which were only 2.2% in the MHLW database, compared to 8.4% in the YCU registry (Table 1). We suspect that there is some relationship between GI and vascular lesions, but we are unable to establish that point in this study because of the different periods of observation in two cohorts. Therefore, it is necessary to validate in a population of patients with similar disease duration.

There is accumulating evidence that clinical presentations of BD are different among ethnic groups, suggesting that each ethnic has a different clustering pattern [28]. The acne-arthritis-enthesitis cluster in Turkey, which likely belongs on the spectrum of seronegative spondyloarthritis [28], was not apparent in our population, though the clinical manifestations were not sufficiently assessed for comparison. This is partly due to the lower prevalence of enthesitis, spondyloarthritis, and HLA-B27 positivity in Japan than in Europe [45]. Both mucocutaneous manifestations and arthritis were enriched in cluster 1 of the YCU registry and cluster E of the MHLW database; these clusters are possible counterparts to the acne-arthritis-enthesitis cluster in Turkey. Further studies with precise phenotypic data are necessary to assess whether the acne-arthritis-enthesitis cluster exists in Japan; moreover, international comparative studies on BD cluster patterns might shed light on ethnic and geographic variations in the disease.

To establish precision medicine, it is important to focus more on genetic contributions rather than environmental factors. We previously reported multiple BD susceptibility loci through genome-wide association studies (GWAS) such as HLA-A26, *IL23R*, and others [46–49]. As shown in Tables 2 and 3, HLA-B51 positivity was significantly less prevalent in cluster 2 (YCU) and cluster A (MHLW) than in other clusters, suggesting that the GI cluster is associated with a distinct genetic background [50]. Identification of the diverse genetic characteristics of the intestinal variant will facilitate its management as a separate disease entity [51]. Moreover, it was possible that autoinflammatory disease (AID) associated with single gene mutation had been diagnosed as BD. For example, “haploinsufficiency of A20” which is caused by *TNFAIP3* mutations has a phenotype similar to that of patients in the GI cluster of BD [52].

Several studies have attempted to subcategorize BD based on clinical manifestations [26, 27, 53]; however, a patient’s natural disease course [13, 17] as well as treatment-related disease modification may prevent the emergence of other phenotypes [54]. Thus, because phenotype-based analysis alone is not necessarily sufficient, the inclusion of genetic analysis would be more instructive.

Several limitations should be noted. First, our results are not necessarily be generalizable to other countries because most of the patients were Japanese who had relatively unique and homogenous genetic backgrounds. Second, selection bias was unavoidable because of the study design; however, we minimized this bias by evaluating two distinct patient populations with unique profiles. Finally, there are only a few reports of standardized index for severity of BD [55, 56]. Therefore, we would like to investigate this point by establishing a large-scale BD patient’s registry in Japan.

**Fig. 2:**
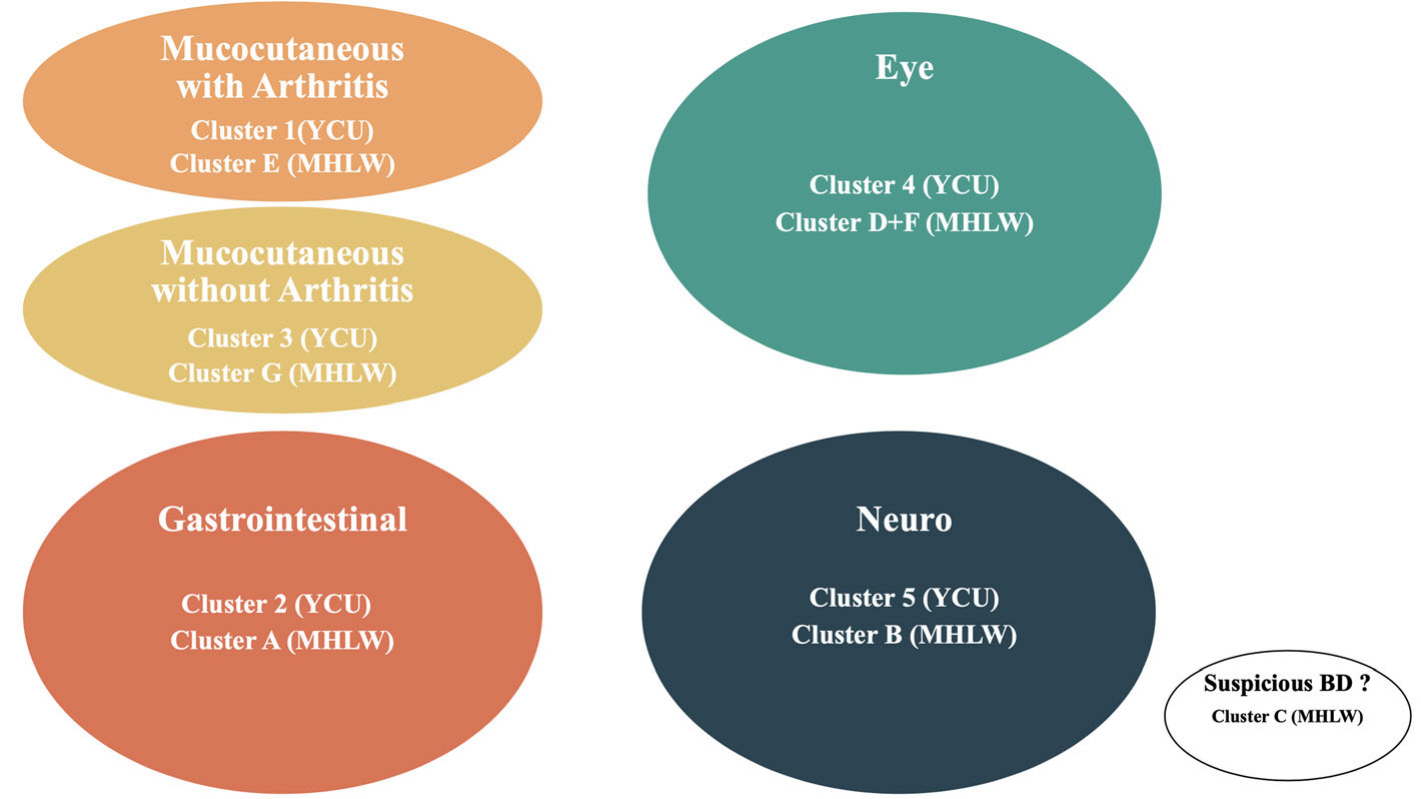
Proposing Behçet’s disease clusters based on the present and previous studies

Based on our current findings and our clinical experience, we propose four distinct clinical clusters namely “mucocutaneous,” which consists of either with or without arthritis, “neuro,” “GI,” and “eye”(Fig. 2). To establish this concept, it is important to further accumulate future data by analyzing genetic and biological samples. A multidisciplinary approach is useful for the early detection of clinical clusters. Ideally, treatment should be optimized for individual clinical subtypes, as the prognosis varies between clusters.

## Conclusion

We identified independent clinical clusters among BD patients in Japan and found that the proportion of each cluster varied over time.

## Supplementary Information

**Additional file 1.** Supplementary file.

## Data Availability

The datasets used and analyzed during the current study are available from the corresponding author on a reasonable request.

## Abbreviations

AID: Autoinflammatory disease
BD: Behçet’s disease
BDRC: Behçet’s Disease Research Committee
GI: Gastrointestinal
GWAS: Genome-wide association studies
HA20: Haploinsufficiency of A20
HLA: Human leukocyte antigen
ISG: International Study Group
ITR-ICBD: International Team for the Revision of the International Criteria for Behçet’s Disease
MHLW: Ministry of Health, Labour and Welfare
TNF: Tumor necrosis factor
YCU: Yokohama City University

## References

[1] Sakane T, Takeno M, Suzuki N, Inaba G (1999). Behcet’s disease. N Engl J Med.

[2] Yazici H, Seyahi E, Hatemi G, Yazici Y (2018). Behcet syndrome: a contemporary view. Nat Rev Rheumatol.

[3] de Menthon M, Lavalley MP, Maldini C, Guillevin L, Mahr A (2009). HLA-B51/B5 and the risk of Behcet’s disease: a systematic review and meta-analysis of case-control genetic association studies. Arthritis Rheum.

[4] Ono S, Aoki K, Sugiura S, Nakayama E, Itakura K (1973). Letter: HL-A5 and Behcet’s disease. Lancet.

[5] Ombrello MJ, Kirino Y, de Bakker PI, Gul A, Kastner DL, Remmers EF (2014). Behcet disease-associated MHC class I residues implicate antigen binding and regulation of cell-mediated cytotoxicity. Proc Natl Acad Sci U S A.

[6] Hatemi G, Christensen R, Bang D, Bodaghi B, Celik AF, Fortune F, Gaudric J, Gul A, Kotter I, Leccese P (2018). 2018 update of the EULAR recommendations for the management of Behcet’s syndrome. Ann Rheum Dis.

[7] Desbois AC, Vallet H, Domont F, Comarmond C, Cacoub P, Saadoun D (2017). Management of severe complications in Behcet’s disease with TNF inhibitors. Expert Opin Biol Ther.

[8] Yazici H, Tuzun Y, Pazarli H, Yurdakul S, Ozyazgan Y, Ozdogan H, Serdaroglu S, Ersanli M, Ulku BY, Muftuoglu AU (1984). Influence of age of onset and patient’s sex on the prevalence and severity of manifestations of Behcet’s syndrome. Ann Rheum Dis.

[9] Ishido T, Horita N, Takeuchi M, Kawagoe T, Shibuya E, Yamane T, Hayashi T, Meguro A, Ishido M, Minegishi K (2017). Clinical manifestations of Behcet’s disease depending on sex and age: results from Japanese nationwide registration. Rheumatology (Oxford).

[10] Kone-Paut I, Shahram F, Darce-Bello M, Cantarini L, Cimaz R, Gattorno M, Anton J, Hofer M, Chkirate B, Bouayed K (2016). Consensus classification criteria for paediatric Behcet’s disease from a prospective observational cohort: PEDBD. Ann Rheum Dis.

[11] Ishigatsubo Y, Takeno M, Ishigatsubo Y (2015). Overview. Behçet’s disease: from genetics to therapies.

[12] Ideguchi H, Suda A, Takeno M, Miyagi R, Ueda A, Ohno S, Ishigatsubo Y (2014). Gastrointestinal manifestations of Behcet’s disease in Japan: a study of 43 patients. Rheumatol Int.

[13] Kirino Y, Ideguchi H, Takeno M, Suda A, Higashitani K, Kunishita Y, Takase-Minegishi K, Tamura M, Watanabe T, Asami Y (2016). Continuous evolution of clinical phenotype in 578 Japanese patients with Behcet’s disease: a retrospective observational study. Arthritis Res Ther.

[14] Kim DY, Choi MJ, Cho S, Kim DW, Bang D (2014). Changing clinical expression of Behcet disease in Korea during three decades (1983–2012): chronological analysis of 3674 hospital-based patients. Br J Dermatol.

[15] Sarica-Kucukoglu R, Akdag-Kose A, Kayabal IM, Yazganoglu KD, Disci R, Erzengin D, Azizlerli G (2006). Vascular involvement in Behcet’s disease: a retrospective analysis of 2319 cases. Int J Dermatol.

[16] Davatchi F, Shahram F, Chams-Davatchi C, Shams H, Abdolahi BS, Nadji A, Faezi T, Akhlaghi M, Ghodsi Z, Karimi N (2019). Behcet’s disease in Iran: analysis of 7641 cases. Mod Rheumatol.

[17] Ideguchi H, Suda A, Takeno M, Ueda A, Ohno S, Ishigatsubo Y. Behcet disease: evolution of clinical manifestations. Medicine (Baltimore). 2011;90(2):125–3. 10.1097/MD.0b013e318211bf28 21358436

[18] Mizuki Y, Horita N, Horie Y, Takeuchi M, Ishido T, Mizuki R, Kawagoe T, Shibuya E, Yuda K, Ishido M (2020). The influence of HLA-B51 on clinical manifestations among Japanese patients with Behcet’s disease: a nationwide survey. Mod Rheumatol.

[19] Kuwahara E, Murakami Y, Nakamura T, Inoue N, Nagahori M, Matsui T, Watanabe M, Suzuki Y, Nishiwaki Y (2017). Factors associated with exacerbation of newly diagnosed mild ulcerative colitis based on a nationwide registry in Japan. J Gastroenterol.

[20] Ng WK, Wong SH, Ng SC (2016). Changing epidemiological trends of inflammatory bowel disease in Asia. Intest Res.

[21] Maldini C, Lavalley MP, Cheminant M, de Menthon M, Mahr A (2012). Relationships of HLA-B51 or B5 genotype with Behcet’s disease clinical characteristics: systematic review and meta-analyses of observational studies. Rheumatology (Oxford).

[22] Suwa A, Horita N, Ishido T, Takeuchi M, Kawagoe T, Shibuya E, Yamane T, Hayashi T, Meguro A, Ishido M (2019). The ocular involvement did not accompany with the genital ulcer or the gastrointestinal symptoms at the early stage of Behcet’s disease. Mod Rheumatol.

[23] Kroot EJ, de Jong BA, van Leeuwen MA, Swinkels H, van den Hoogen FH, van't Hof M, van de Putte LB, van Rijswijk MH, van Venrooij WJ, van Riel PL (2000). The prognostic value of anti-cyclic citrullinated peptide antibody in patients with recent-onset rheumatoid arthritis. Arthritis Rheum.

[24] Ding B, Padyukov L, Lundstrom E, Seielstad M, Plenge RM, Oksenberg JR, Gregersen PK, Alfredsson L, Klareskog L (2009). Different patterns of associations with anti-citrullinated protein antibody-positive and anti-citrullinated protein antibody-negative rheumatoid arthritis in the extended major histocompatibility complex region. Arthritis Rheum.

[25] Holoshitz J (2010). The rheumatoid arthritis HLA-DRB1 shared epitope. Curr Opin Rheumatol.

[26] Hatemi G, Fresko I, Tascilar K, Yazici H (2008). Increased enthesopathy among Behcet’s syndrome patients with acne and arthritis: an ultrasonography study. Arthritis Rheum.

[27] Jun Hayashi CT, Sugano Y, Nonaka N, Enomoto M, Ishii O, Nakamura T, Matsuda T, Sugimmori H (2005). Study on clinical manifestations of Behcet’s disease by cluster analysis. Clin Rheumatol Relat Res.

[28] Yazici H, Ugurlu S, Seyahi E (2012). Behcet syndrome: is it one condition?. Clin Rev Allergy Immunol.

[29] Seyahi E (2019). Phenotypes in Behcet’s syndrome. Intern Emerg Med.

[30] Karaca M, Hatemi G, Sut N, Yazici H (2012). The papulopustular lesion/arthritis cluster of Behcet’s syndrome also clusters in families. Rheumatology (Oxford).

[31] Mizushima Y, Inaba G, Mimura Y (1987). Guide for the diagnosis of Behçet’s disease. Report of Behçet’s Disease Research Committee, Japan.

[32] Criteria for diagnosis of Behcet’s disease (1990). International study group for Behcet’s disease. Lancet.

[33] The International Criteria for Behcet's Disease (2014). (ICBD): a collaborative study of 27 countries on the sensitivity and specificity of the new criteria. J Eur Acad Dermatol Venereol.

[34] Caliński T, J Harabasz J. A dendrite method for cluster analysis. Communications in Statistics. 1974;3(1):1–27.

[35] Hartigan JA. Clustering analysis. Wiley; 1975.

[36] Krzanowski WJ, Lai YT (1985). A criterion for determining the number of clusters in a data set. Biometrics..

[37] Hamuryudan V, Ozyazgan Y, Hizli N, Mat C, Yurdakul S, Tuzun Y, Senocak M, Yazici H (1997). Azathioprine in Behcet’s syndrome: effects on long-term prognosis. Arthritis Rheum..

[38] Nakahara H, Kaburaki T, Tanaka R, Yoshida A, Takamoto M, Kawata M, Fujino Y, Kawashima H, Aihara M (2020). Comparisons of clinical features in Japanese patients with Behce’s uveitis treated in the 1990s and the 2000s. Ocul Immunol Inflamm..

[39] Aramaki K, Kikuchi H, Hirohata S (2007). HLA-B51 and cigarette smoking as risk factors for chronic progressive neurological manifestations in Behcet’s disease. Mod Rheumatol..

[40] Malek Mahdavi A, Khabbazi A, Yaaghoobian B, Ghojazadeh M, Agamohammadi R, Kheyrollahiyan A, Rashtchizadeh N (2019). Cigarette smoking and risk of Behcet’s disease: a propensity score matching analysis. Mod Rheumatol..

[41] Lee YB, Lee JH, Lee SY, Yu DS, Han KD, Park YG (2019). Association between smoking and Behcet’s disease: a nationwide population-based study in Korea. J Eur Acad Dermatol Venereol..

[42] Rizvi SW, McGrath H (2001). The therapeutic effect of cigarette smoking on oral/genital aphthosis and other manifestations of Behcet’s disease. Clin Exp Rheumatol..

[43] Direskeneli H, Mumcu G (2010). A possible decline in the incidence and severity of Behcet’s disease: implications for an infectious etiology and oral health. Clin Exp Rheumatol..

[44] Consolandi C, Turroni S, Emmi G, Severgnini M, Fiori J, Peano C, Biagi E, Grassi A, Rampelli S, Silvestri E (2015). Behcet’s syndrome patients exhibit specific microbiome signature. Autoimmun Rev..

[45] Kishimoto M, Yoshida K, Ichikawa N, Inoue H, Kaneko Y, Kawasaki T, Matsui K, Morita M, Suda M, Tada K (2019). Clinical characteristics of patients with spondyloarthritis in Japan in comparison with other regions of the world. J Rheumatol..

[46] Remmers EF, Cosan F, Kirino Y, Ombrello MJ, Abaci N, Satorius C, Le JM, Yang B, Korman BD, Cakiris A (2010). Genome-wide association study identifies variants in the MHC class I, IL10, and IL23R-IL12RB2 regions associated with Behcet’s disease. Nat Genet..

[47] Kirino Y, Bertsias G, Ishigatsubo Y, Mizuki N, Tugal-Tutkun I, Seyahi E, Ozyazgan Y, Sacli FS, Erer B, Inoko H (2013). Genome-wide association analysis identifies new susceptibility loci for Behcet’s disease and epistasis between HLA-B*51 and ERAP1. Nat Genet..

[48] Mizuki N, Meguro A, Ota M, Ohno S, Shiota T, Kawagoe T, Ito N, Kera J, Okada E, Yatsu K, et al. Genome-wide association studies identify IL23R-IL12RB2 and IL10 as Behcet’s disease susceptibility loci. Nat Genet. 2010;42(8):703–6. 10.1038/ng.624 20622879

[49] Gul A (2011). Genome-wide association studies in Behcet’s disease: expectations and promises. Clin Exp Rheumatol..

[50] Kim SW, Jung YS, Ahn JB, Shin ES, Jang HW, Lee HJ, Il Kim T, Kim DY, Bang D, Kim WH (2017). Identification of genetic susceptibility loci for intestinal Behcet’s disease. Sci Rep..

[51] Kirino Y, Nakajima H (2019). Clinical and genetic aspects of Behcet’s disease in Japan. Intern Med..

[52] Tsuchida N, Kirino Y, Soejima Y, Onodera M, Arai K, Tamura E, Ishikawa T, Kawai T, Uchiyama T, Nomura S (2019). Haploinsufficiency of A20 caused by a novel nonsense variant or entire deletion of TNFAIP3 is clinically distinct from Behcet’s disease. Arthritis Res Ther..

[53] Arida A, Vaiopoulos G, Markomichelakis N, Kaklamanis P, Sfikakis PP. Are clusters of patients with distinct clinical expression present in Behcet’s disease? Clin Exp Rheumatol. 2009;27(2 Suppl 53):S48–51. 19796533

[54] Yazici H, Pazarli H, Barnes CG, Tuzun Y, Ozyazgan Y, Silman A, Serdaroglu S, Oguz V, Yurdakul S, Lovatt GE (1990). A controlled trial of azathioprine in Behcet’s syndrome. N Engl J Med..

[55] Krause I, Rosen Y, Kaplan I, Milo G, Guedj D, Molad Y, Weinberger A. Recurrent aphthous stomatitis in Behçet’s disease: clinical features and correlation with systemic disease expression and severity. J Oral Pathol Med. 1999;28(5):193–6. 10.1111/j.1600-0714.1999.tb02023.x 10226940

[56] Krause I, Mader R, Sulkes J, Paul M, Uziel Y, Adawi M, Weinberger A. Behçet’s disease in Israel: the influence of ethnic origin on disease expression and severity. J Rheumatol. 2001;28(5):1033–6. 11361184

