# Supplementary material for: Changes in the proportion of clinical clusters contribute to the phenotypic evolution of Behçet’s disease in Japan
Source: Arthritis Res Ther. 2021 Feb 1;23:49. doi: 10.1186/s13075-020-02406-6 (PMC7851921; doi:10.1186/s13075-020-02406-6)
Supplement: Supplementary file 1 — Additional file 1. Supplementary file. [file 13075_2020_2406_MOESM1_ESM.docx]

**Supplementary data**

**Supplementary Figure 1**

Flow diagram of BD patients participating in the study.


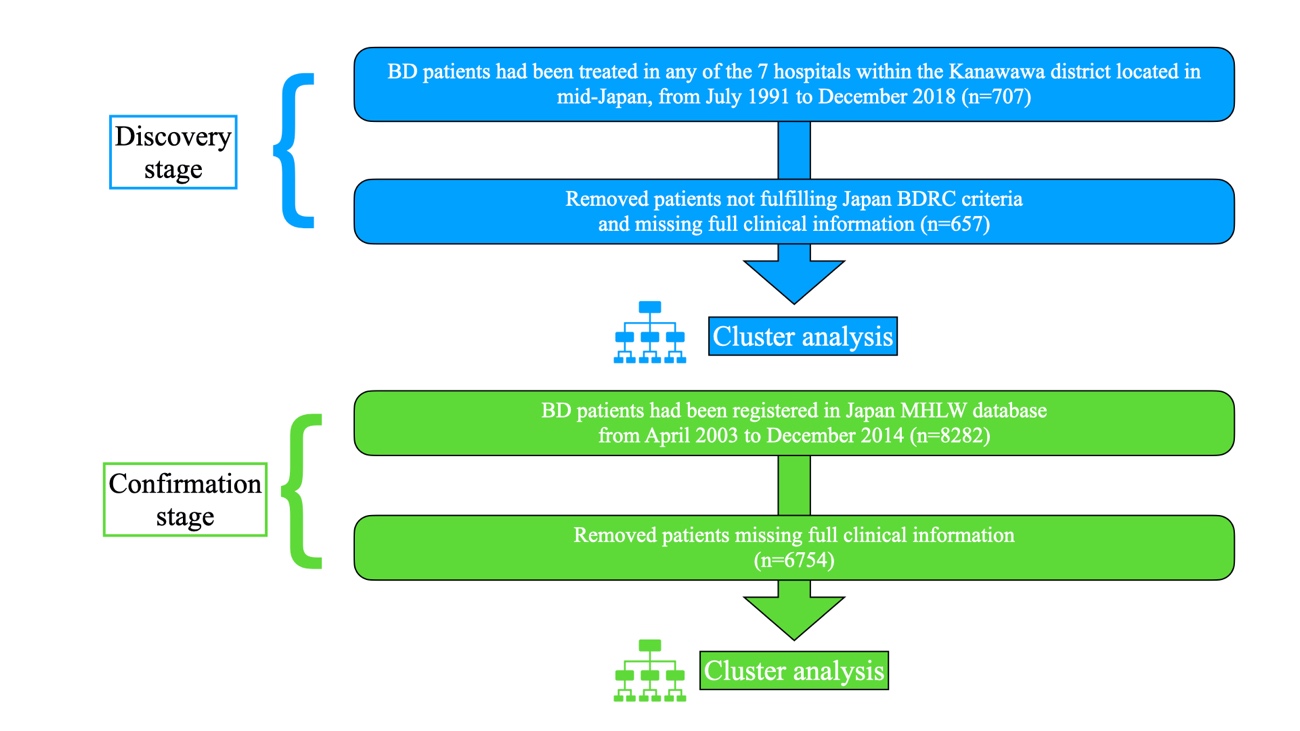


**Supplementary Figure 2**

Dendrogram showing cluster analysis of YCU registry (a) and MHLW registry (b).


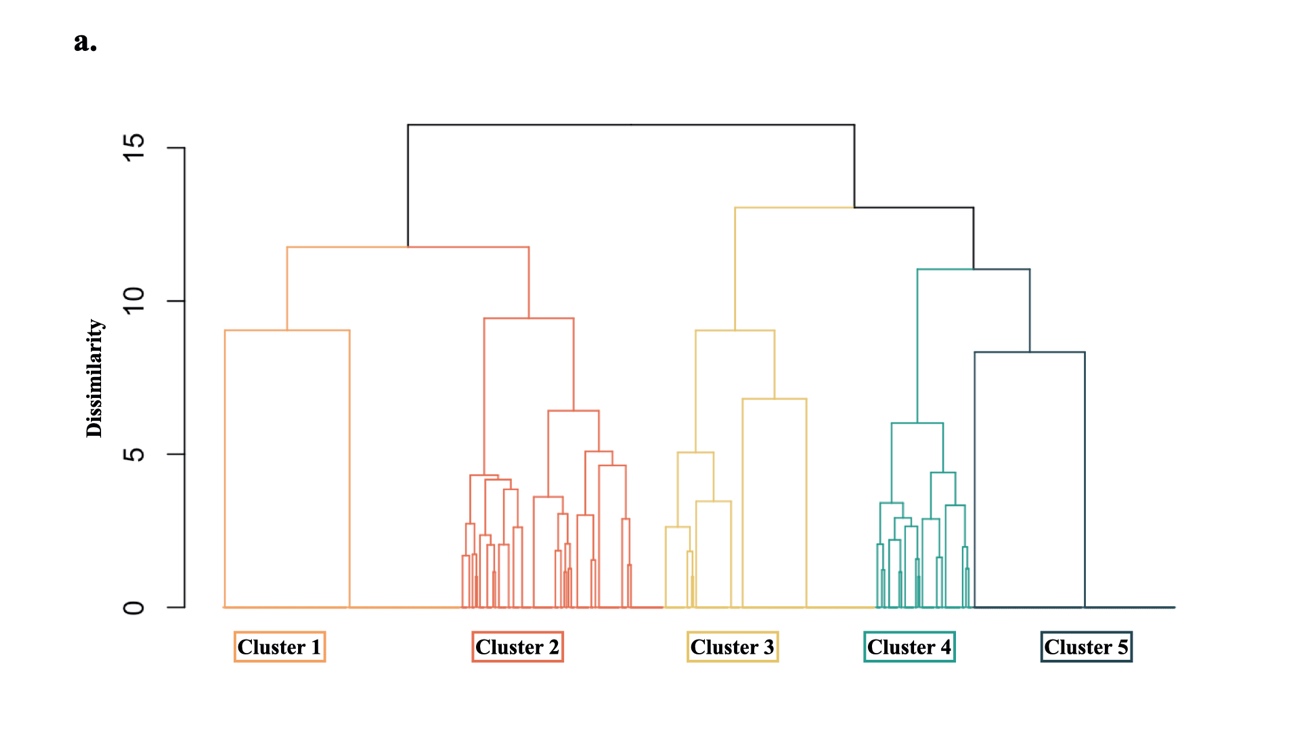


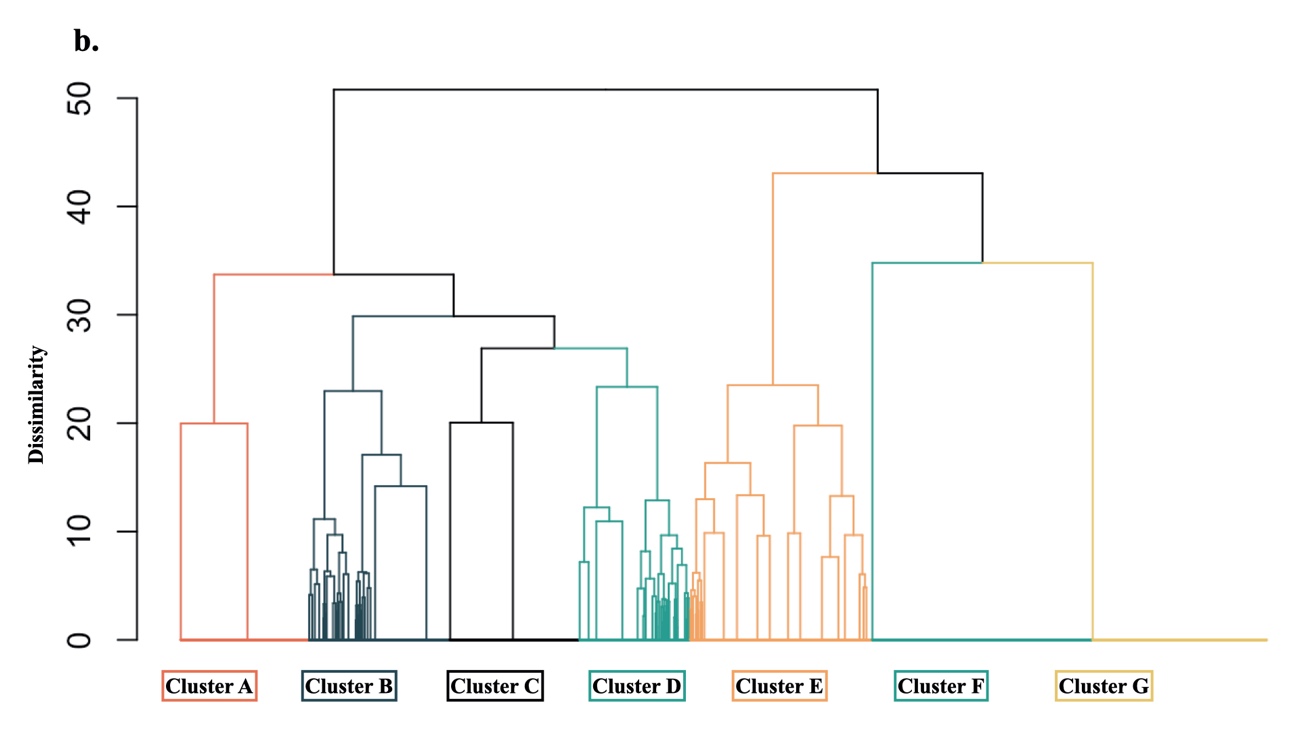


YCU: Yokohama City University, MHLW: Ministry of Health, Labour and Welfare

**Supplementary Table 1 Statistical measures to show the optimal number of clusters**

| **YCU registry** |  |  |  |  |
| --- | --- | --- | --- | --- |
| Number of clusters | CH | H | diffH | KL |
| 2 | 134.9674 | 107.75558 | 27.2118266 | 0.2841442 |
| 3 | 132.3604 | 100.83488 | 6.9206986 | 0.7587832 |
| 4 | 135.322 | 102.68039 | -1.8455133 | 0.859112 |
| 5 | 142.9651 | 84.66658 | 18.0138136 | 1.2826014 |
| 6 | 145.982 | 88.20471 | -3.5381317 | 0.9389083 |
| 7 | 152.6483 | 101.77691 | -13.5722009 | 0.8733478 |
| 8 | 165.6668 | 99.6982 | 2.0787169 | 1.1060985 |
| 9 | 179.4656 | 74.02205 | 25.6761442 | 1.5292897 |
| 10 | 185.7262 | 73.07 | 0.9520525 | 1.0685848 |
| **MHLW registry** |  |  |  |  |
| Number of clusters | CH | H | diffH | KL |
| 2 | 1406.153 | 1188.8901 | 217.26271 | 0.27138 |
| 3 | 1421.215 | 876.8914 | 311.99878 | 1.2808297 |
| 4 | 1362.702 | 938.0086 | -61.11723 | 0.7187193 |
| 5 | 1398.402 | 825.7467 | 112.26191 | 1.1438784 |
| 6 | 1420.582 | 743.782 | 81.96465 | 1.1323097 |
| 7 | 1438.09 | 620.1411 | 123.64096 | 1.2856374 |
| 8 | 1434.355 | 672.5446 | -52.40355 | 0.8831711 |
| 9 | 1464.066 | 720.1584 | -47.61376 | 0.936894 |
| 10 | 1520.165 | 597.4899 | 122.66852 | 1.3167707 |

YCU: Yokohama City University, MHLW: Ministry of Health, Labour and Welfare

**Supplementary Table 2 Characteristics of BD patients’ clusters in YCU registry excluding patients not fulfilling ISG criteria**

| **Characteristics** | **Total (n=583)** | **Cluster 1 (n=164)** | **Cluster 2 (n=107)** | **Cluster 3 (n=140)** | **Cluster 4 (n=113)** | **Cluster 5 (n=59)** |
| --- | --- | --- | --- | --- | --- | --- |
| Skin involvement, n (%) | 559 (95.9) | 164 (100.0) | 107 (100.0) | 140 (100.0) | 93 (82.3) | 55 (93.2) |
| Eye involvement, n (%) | 351 (60.2) | 78 (47.6) | 44 (41.1) | 76 (54.3) | 113 (100.0) | 40 (67.8) |
| Genital ulcer, n (%) | 463 (79.4) | 164 (100.0) | 97 (90.7) | 140 (100.0) | 19 (16.8) | 43 (72.9) |
| Arthritis, n (%) | 304 (52.1) | 164 (100.0) | 65 (60.7) | 0 (0.0) | 48 (42.5) | 27 (45.8) |
| Gastrointestinal involvement, n (%) | 85 (14.6) | 0 (0.0) | 79 (73.8) | 0 (0.0) | 0 (0.0) | 6 (10.2) |
| Vascular involvement, n (%) | 42 (7.2) | 0 (0.0) | 41 (38.3) | 0 (0.0) | 0 (0.0) | 1 (1.7) |
| Neurological involvement, n (%) | 59 (10.1) | 0 (0.0) | 1 (0.9) | 0 (0.0) | 0 (0.0) | 58 (98.3) |
| Sex: Female/Male, n (%) | 344/239 (59.0/41.0) | 124/40 (75.6/24.4) | 67/40 (62.6/37.4) | 90/50 (64.3/35.7) | 33/80 (29.2/70.8) | 30/29 (50.8/49.2) |
| Age at onset (years, mean±SD) | 35.97±11.91 | 37.37±12.25 | 34.35±12.57 | 33.59±10.25 | 39.10±12.59 | 34.62±10.50 |
| Observation period (years, mean±SD) | 14.43±12.07 | 16.33±12.07 | 15.21±13.11 | 13.27±12.53 | 11.27±10.16 | 16.51±11.11 |
| Pathergy test, n (%) | 70/159 (44.0) | 27/66 (40.9) | 6/13 (31.6) | 21/34 (61.8) | 10/26 (38.5) | 6/14 (42.9) |
| HLA-B51, n (%) | 196/403 (48.6) | 58/114 (50.9) | 26/72 (36.1) | 49/94 (52.1) | 3976 (51.3) | 24/47 (51.1) |
| Fulfilling ITR-ICBD criteria, n (%) | 583 (100.0) | 164 (100.0) | 107 (100.0) | 140 (100.0) | 113 (100.0) | 59 (100.0) |
| Colchicine, n (%) | 336/470 (71.5) | 73/115 (63.5) | 63/82 (76.8) | 83/128 (64.8) | 76/95 (80.0) | 41/50 (82.0) |
| Glucocorticoids, n (%) | 210/470 (44.7) | 40/115 (34.8) | 57/82 (69.5) | 44/128 (34.4) | 30/95 (31.6) | 39/50 (78.0) |
| Maximum Prednisolone dose (mg/day, mean±SD) | 11.25±17.31 | 5.64±11.15 | 18.38±18.50 | 6.81±12.71 | 7.50±14.08 | 31.70±23.73 |
| Methylprednisolone pulse therapy, n (%) | 32/470 (6.8) | 2/115 (1.7) | 8/82 (9.8) | 2/128 (1.6) | 2/95 (2.1) | 18/50 (36.0) |
| Immunosuppressants, n (%) | 177/470 (37.7) | 17/115 (14.8) | 62/82 (75.6) | 30/128 (23.4) | 34/95 (35.8) | 34/50 (68.0) |
| Biologics, n (%) | 79 (13.6) | 9 (5.5) | 19 (17.8) | 15 (10.7) | 23 (20.4) | 13 (22.0) |
| Time from diagnosis to biologics (years, mean±SD) | 7.08±9.03 | 4.86±6.90 | 8.24±11.92 | 10.07±10.20 | 3.92±5.02 | 8.90±8.65 |
| Hospitalization, n (%) | 206/470 (43.8) | 35/115 (30.4) | 56/82 (68.3) | 43/128 (33.6) | 35/95 (36.8) | 37/50 (74.0) |

ISG: International Study Group, ITR-ICBD: International Team for the Revision of the International Criteria for Behçet’s Disease.

**Supplementary Table 3 Characteristics of BD patients’ clusters in YCU registry excluding patients not fulfilling ITR-ICBD criteria**

| **Characteristics** | **Total (n=645)** | **Cluster 1 (n=164)** | **Cluster 2 (n=128)** | **Cluster 3 (n=140)** | **Cluster 4 (n=146)** | **Cluster 5 (n=67)** |
| --- | --- | --- | --- | --- | --- | --- |
| Oral ulcer, n (%) | 643 (99.7) | 164 (100.0) | 128 (100.0) | 140 (100.0) | 144 (98.6) | 67 (100.0) |
| Skin involvement, n (%) | 573 (88.8) | 164 (100.0) | 118 (92.2) | 140 (100.0) | 93 (63.7) | 58 (86.6) |
| Eye involvement, n (%) | 391 (60.6) | 78 (47.6) | 47 (36.7) | 76 (54.3) | 146 (100.0) | 44 (65.7) |
| Genital ulcer, n (%) | 473 (73.3) | 164 (100.0) | 104 (81.2) | 140 (100.0) | 21 (14.4) | 44 (65.7) |
| Arthritis, n (%) | 334 (51.8) | 164 (100.0) | 82 (64.1) | 0 (0.0) | 57 (39.0) | 31 (46.3) |
| Gastrointestinal involvement, n (%) | 101 (15.7) | 0 (0.0) | 94 (73.4) | 0 (0.0) | 0 (0.0) | 7 (10.4) |
| Vascular involvement, n (%) | 55 (8.5) | 0 (0.0) | 54 (42.2) | 0 (0.0) | 0 (0.0) | 1 (1.5) |
| Neurological involvement, n (%) | 67 (10.4) | 0 (0.0) | 1 (0.8) | 0 (0.0) | 0 (0.0) | 66 (98.5) |
| Sex: Female/Male, n (%) | 366/279 (56.7/43.3) | 124/40 (75.6/24.4) | 74/54 (57.8/42.2) | 90/50 (64.3/35.7) | 46/100 (31.5/68.5) | 32/35 (47.8/52.2) |
| Age at onset (years, mean±SD) | 36.63±12.25 | 37.37±12.25 | 35.59±13.32 | 33.59±10.25 | 40.48±12.84 | 34.79±10.43 |
| Observation period (years, mean±SD) | 13.80±11.83 | 16.33±12.07 | 14.09±12.74 | 13.27±12.53 | 10.16±9.44 | 16.08±10.70 |
| Pathergy test, n (%) | 73/169 (43.2) | 27/66 (40.9) | 6/21 (28.6) | 21/34 (61.8) | 13/32 (40.6) | 6/16 (37.5) |
| HLA-B51, n (%) | 212/444 (47.7) | 58/114 (50.9) | 27/83 (32.5) | 49/94 (52.1) | 49/98 (50.0) | 29/55 (52.7) |
| Fulfilling ITR-ICBD criteria, n (%) | 583 (90.4) | 164 (100.0) | 107 (83.6) | 140 (100.0) | 113 (77.4) | 59 (88.1) |
| Colchicine, n (%) | 371/518 (71.6) | 73/115 (63.5) | 73/93 (78.5) | 83/128 (64.8) | 96/125 (76.8) | 46/57 (80.7) |
| Glucocorticoids, n (%) | 233/518 (45.0) | 40/115 (34.8) | 62/93 (66.7) | 44/128 (34.4) | 42/125 (33.6) | 45/57 (78.9) |
| Maximum Prednisolone dose (mg/day, mean±SD) | 11.62±17.62 | 5.64±11.15 | 18.46±19.36 | 6.81±12.71 | 8.16±14.24 | 31.81±23.64 |
| Methylprednisolone pulse therapy, n (%) | 36/518 (6.9) | 2/115 (1.7) | 9/93 (9.7) | 2/128 (1.6) | 4/125 (3.2) | 19/57 (33.3) |
| Immunosuppressants, n (%) | 200/518 (38.6) | 17/115 (14.8) | 68/93 (73.1) | 30/128 (23.4) | 47/125 (37.6) | 38/57 (66.7) |
| Biologics, n (%) | 93 (14.4) | 9 (5.5) | 21 (16.4) | 15 (10.7) | 34 (23.3) | 14 (20.9) |
| Time from diagnosis to biologics (years, mean±SD) | 6.25±8.61 | 4.86±6.90 | 7.69±11.45 | 10.07±10.20 | 2.96±4.46 | 8.54±8.42 |
| Hospitalization, n (%) | 236/518 (45.6) | 35/115 (30.4) | 66/93 (71.0) | 43/128 (33.6) | 49/125 (39.2) | 43/57 (75.4) |

ISG: International Study Group, ITR-ICBD: International Team for the Revision of the International Criteria for Behçet’s Disease.

**Supplementary Table 4 Chronological comparison of characteristics of BD patients in YCU registry**

| **Characteristics** | **Total (n=657)** | **before 1999 (n=330)** | **2000-2009 (n=199)** | **after 2010 (n=128)** | **p** |
| --- | --- | --- | --- | --- | --- |
| Sex: Female/Male, n (%) | 372/285 (56.6/43.4) | 191/139 (57.9/42.1) | 111/88 (55.8/44.2) | 70/58 (54.7/45.3) | 0.793 |
| Age of diagnosis (mean±SD) | 36.58±12.29 | 36.34 (11.65) | 35.72 (12.34) | 38.51 (13.64) | 0.119 |
| Observation period (mean±SD) | 13.72±11.79 | 20.92 (12.26) | 8.36 (4.92) | 3.51 (2.57) | <0.001 |
| Oral ulcer, n (%) | 653 (99.4) | 328 (99.4) | 198 (99.5) | 127 (99.2) | 0.951 |
| Skin involvement, n (%) | 585 (89.0) | 299 (90.6) | 178 (89.4) | 108 (84.4) | 0.156 |
| Eye involvement, n (%) | 392 (59.7) | 217 (65.8) | 109 (54.8) | 66 (51.6) | 0.005 |
| Genital ulcer, n (%) | 474 (72.1) | 254 (77.0) | 141 (70.9) | 79 (61.7) | 0.004 |
| Arthritis, n (%) | 346 (52.7) | 172 (52.1) | 108 (54.3) | 66 (51.6) | 0.857 |
| Epididymitis, n/available data number (%) | 15/285 (5.3) | 10/139 (7.2) | 3/88 (3.4) | 2/58 (3.4) | 0.363 |
| Gastrointestinal involvement, n (%) | 113 (17.2) | 48 (14.5) | 33 (16.6) | 32 (25.0) | 0.028 |
| Vascular involvement, n (%) | 55 (8.4) | 29 (8.8) | 16 (8.0) | 10 (7.8) | 0.925 |
| Neurological involvement, n (%) | 67 (10.2) | 40 (12.1) | 18 (9.0) | 9 (7.0) | 0.221 |
| Pathergy test, n/available data number (%) | 74/170 (43.5) | 55/112 (49.1) | 12/38 (31.6) | 7/20 (35.0) | 0.121 |
| HLA-B51, n/available data number (%) | 214/449 (47.7) | 120/228 (52.6) | 62/124 (50.0) | 32/97 (33.0) | 0.004 |
| Fulfilling ISG criteria, n (%) | 583 (88.7) | 307 (93.0) | 173 (86.9) | 103 (80.5) | <0.001 |
| Fulfilling ITR-ICBD criteria, n (%) | 645 (98.2) | 325 (98.5) | 195 (98.0) | 125 (97.7) | 0.816 |
| Smoking, n/available data number (%) | 233/455 (51.2) | 104/218 (47.7) | 63/123 (51.2) | 66/114 (57.9) | 0.211 |
| Hospitalization, n/available data number (%) | 239/522 (45.8) | 116/267 (43.4) | 58/140 (41.4) | 65/115 (56.5) | 0.03 |
| Blindness, n/available data number (%) | 98/582 (16.8) | 84/287 (29.3) | 10/173 (5.8) | 4/122 (3.3) | <0.001 |
| Colchicine, n (%) | 375/522 (71.8) | 166/267 (62.2) | 112/140 (80.0) | 97/115 (84.3) | <0.001 |
| Glucocorticoid, n (%) | 236/522 (45.2) | 119267 (44.6) | 73/140 (52.1) | 44/115 (38.3) | 0.082 |
| Maximum Prednisolone dose (mg/day, mean±SD) | 11.75±17.76 | 11.78 (18.44) | 11.80 (16.00) | 11.62 (18.34) | 0.996 |
| Methylprednisolone pulse therapy, n/available data number (%) | 36/522 (6.9) | 18/267 (6.7) | 9/140 (6.4) | 9/115 (7.8) | 0.899 |
| Immunosuppressant therapy, n/available data number (%) | 203/522 (38.9) | 104/267 (39.0) | 52/140 (37.1) | 47/115 (40.9) | 0.831 |
| Biologics, n (%) | 95 (14.5) | 24 (7.3) | 32 (16.1) | 39 (30.5) | <0.001 |
| Time from diagnosis to biologics (years, mean±SD) | 6.22±8.52 | 16.47 (10.55) | 4.33 (3.81) | 1.22 (1.76) | <0.001 |
| BDRC: Behçet's Disease Research Committee, ISG: International Study Group, ITR-ICBD: International Team for the Revision of the International Criteria for Behçet’s Disease, N/A: not available. | | | | | |
